# Evaluation of accessibility and equity to hospitals by public transport: evidence from six largest cities of Ohio

**DOI:** 10.1186/s12913-023-09588-0

**Published:** 2023-06-08

**Authors:** M. S. Alam, N. J. Tabassum, A. I. Tokey

**Affiliations:** 1 Department of Geography and Planning, The University of Toledo, Toledo, OH United States; 2 Department of Geography, Ohio State University, Columbus, OH United States

**Keywords:** Accessibility, Equity, Hospitals, Public transport, Vulnerability, Urban planning

## Abstract

**Introduction:**

In the United States, health care has long been viewed as a ‘right,’ and residents of the state of Ohio are no exception. The Ohio Department of Health ensures that this right exists for all residents of Ohio. Socio-spatial characteristics, however, can have an impact on access to health care, particularly among vulnerable groups. This article seeks to measure the spatial accessibility to healthcare services by public transport in the six largest cities of Ohio based on population and to compare the accessibility of healthcare to vulnerable demographic groups. To the authors’ knowledge, this is the first study to analyze the accessibility and equity of hospitals by public transit across different cities in Ohio, allowing the identification of common patterns, difficulties, and knowledge gaps.

**Methodology:**

Using a two-step floating catchment area technique, the spatial accessibility to general medical and surgical hospitals through public transportation was estimated, considering both service-to-population ratios and travel time to these health services. The average accessibility of all census tracts and the average accessibility of the 20% of most susceptible census tracts were determined for each city. Using Spearman’s rank correlation coefficient between accessibility and vulnerability, an indicator was then devised to evaluate vertical equity.

**Findings:**

Within cities (except Cleveland), people of vulnerable census tracts have less access to hospitals via public transportation. These cities (Columbus, Cincinnati, Toledo, Akron, and Dayton) fail in terms of vertical equity and average accessibility. According to this, vulnerable census tracts in these cities have the lowest accessibility levels.

**Conclusion:**

This study emphasizes the issues connected with the suburbanization of poverty in Ohio’s large cities and the need to provide adequate public transportation to reach hospitals on the periphery. In addition, this study shed light on the need for additional empirical research to inform the implementation of guidelines for healthcare accessibility in Ohio. Researchers, planners, and policymakers who want to make healthcare more accessible for everyone should take note of the findings in this study.

## Introduction

Access to medical care is a very complex subject with multiple dimensions. The behavioral model has been utilized for several decades to help identify and characterize these dimensions [1]. The U.S. healthcare system shifted from altruistic decision-making to a posture of caution and fiscal restraint as a result of expanding access to and supply of resources [2]. In the 1970s, the emphasis moved from growing medical care usage to limiting health care costs and establishing procedures to restrict access to health care. In the 1980s and early 1990s, when competing against fee-for-service companies, managed care profit margins increased by double digits [3]. However, its growth slowed over time and managed care firms came under intense scrutiny for allegedly limiting subscribers’ access to necessary services [4]. However, both government and academia have engaged in a substantial dispute about how to interpret this notion of accessibility [5]. Potential and actual access to healthcare has traditionally been classified into two basic groups in academia [5, 6]. A patient’s potential access to healthcare is determined by factors such as the location of available services and the frequency with which those services are actually used [7]. On the contrary, actual access to care refers to those aspects of access that can be seen and measured directly [8]. U.S. healthcare system also defines access as equitable and inequitable. Equitable (as well as inequitable) access is defined based on which variables (age, ethnicity, insurance status, and symptoms) of actual access are most predictive of utilization. Inequitable access exists when social traits and enabling resources, such as race or income, decide who receives medical treatment [9]. Equity of access to medical care is the value judgment that the system is fair or equitable if need-based criteria (rather than enabling resources like insurance coverage or income) are the primary determinants of whether or not care is sought or how much care is sought [4]. While there appears to be a consensus that access to healthcare in terms of location falls under the US Health Act’s definition of accessibility, this does not mean that it is a ‘right.’ Healthcare access is a contentious issue in the United States and developed and developing countries [10, 11]. Amidst the pandemic, the accessibility of healthcare through transit was impeded due to the heightened risk of transmission. However, as time progressed, the situation returned to its original state, thereby increasing the public’s inclination towards utilising public transport as a means of accessing healthcare services [12].

The situation in Ohio is also not satisfactory. Disparities in healthcare access also exist in Ohio [13]. In the United Health Care Foundation’s 2013 study, America’s Health Rankings, Ohio rated forty out of fifty states for total population health [14]. The total population is around 11,756,058 and person under five (5) years is 5.7%, and over 65 years is 17.8% [15]. In 2009, Ohio residents had a life expectancy of 77.8 years, which was lower than the national average of 78.9 years. The rates of diabetes, overweight and obesity, smoking, and infant mortality among Ohio adults are higher than the national averages [16]. In Ohio, the prevalence of diabetes has climbed from 10 to 11.7% of adults in the past year; more than 1 million adults have diabetes. In contrast, the cancer rate in Ohio is lower than the national average [17]. In addition, gaps in health and health access persist throughout the state’s geographic areas, with non-elderly individuals in Ohio’s Appalachian counties more likely to have unmet health needs and believe themselves to be in poor health than their counterparts in more metropolitan counties [18]. It is critical to build a method for quantifying the spatial accessibility of healthcare and analyzing it from an equitable perspective because of the prevalence of spatial determinants that create barriers to accessing health services. In order to quantify the current condition, which is unlikely to be discovered through previous research for the state of Ohio, this article incorporates public transit access to healthcare for the vulnerable population of different cities. The research fills the gaps by exploring transit based healthcare accessibility by considering the vulnerable group of people in Ohio and understanding the equity for all the people to get access to healthcare.

Transportation investments in Ohio have created an unfriendly environment for people experiencing poverty, people with disabilities, and the elderly. People of color are disproportionately disadvantaged by the existing state of transportation due to the high cost of car ownership, the underinvestment in public transit, and the isolation of low-income people and struggling families from services (healthcare, jobs) by major thoroughfares [19]. The state of Ohio invests 99% of its transportation funds on highways, leaving less than 1% for public transportation, ranking it forty-first in the nation [20]. All of the states that spend less on transit than Ohio are more rural, with a population that is 20% smaller than Ohio’s. Nearly 9% of households in Ohio do not own a vehicle [21]. Despite the necessity for public transportation, transit companies in Ohio have been compelled to reduce services and increase rates [20, 22]. Ohio needs a better public transportation system for low-income, middle-income, and elderly and disabled residents to get to and from healthcare institutions.

This paper aims to quantify the spatial accessibility to healthcare services (specifically general medical and surgical hospitals) by public transport in six major cities of Ohio and compare the accessibility to healthcare for vulnerable populations to establish a benchmarking level of access that can be compared across cities. Spatial accessibility to healthcare is measured using a two-step floating catchment area method (2SFCA), which incorporates the spatial relationship between supply (captured by the number of beds) and demand for services (population) as well as competition effects for scarce resources [23]. Some of the most pressing issues in improving access to healthcare in Ohio, especially for vulnerable people, can be identified through the development of accessibility metrics in multiple cities of Ohio. It depicts the considerations that should be made when allocating health and transportation resources.

The objective of this paper is to evaluate the accessibility to hospitals using public transport and understand the nature of equity to access to hospitals by the people, especially for vulnerable groups. The paper is structured into 6 sections. The first section provides an overview of literature based on accessibility and equity in public transport. The next section is to understand the study area and the profile of those areas regarding demography, transport and other metrics. In Sect. 4, the data and methodology were used to calculate accessibility. Section 5 represents the results of the accessibility calculations among these cities and compares the accessibility and equity of those cities to understand the distribution. The last section concludes the paper with proper recommendations.

## Literature review

Accessibility has many definitions considering mobility and other factors and has various approaches to estimating it. It affects the capability to access opportunities, the location of the opportunities, patterns of land use, the quality and affordability of transport options, and people’s ability to use them [24]. According to Litman (2002), accessibility is the ease of reaching goods, services, activities, and destinations, called opportunity [25]. It is also defined from two perspectives: integral and relative accessibility. Relative accessibility refers to how two points on a surface are connected, and integral accessibility refers to how a point is connected with all other points on the same surface [26, 27].

Access to health care is also quantified in terms of accessibility [24, 25, 28–32]. So numerous empirical studies on access to health care and health consequences have been done. Accessibility measurement has often been classified into four categories: travel-cost approach, gravity-based approach, isochron approach, and utility-based approach [31, 33]. Further, it has been grouped broadly into four categories: provider-to-population ratio, distance to the nearest provider, the average distance to a group of providers, and gravity models [6].

The cumulative accessibility concept was presented by Wickstrom in 1971, which quantifies the number of possibilities that may be accessed from a given position in space during a specific time period [34]. It is also calculated using the gravity model, which discounts services based on their distance from the user, meaning that the further a service is from the user, the less it contributes to accessibility [35, 36]. Guagliardo (2004) mentions that average travel time from a point in space to all hospitals is essential [6]. Geographic information systems (GIS) have brought changes in access measurement and conceptualization to incorporate spatial measurement. GIS is one of the gravity model’s fundamental components, which more accurately encompasses the interaction between the provider and the population. Smart card data is used in China to infer the transit based accessibility to demonstrate the use of Big Data in the analysis [37]. Socio-economic activity, like income, has played a part in analyzing health accessibility, especially for low and middle-income countries, which consider a focus in recent studies [38]. Even in recent studies in the USA, the author tried to find out how racial and car ownerships rate affect healthcare accessibility [39]. But our research tries to find out the accessibility to the healthcare of vulnerable groups of people in Ohio cities and find the equity situations based on household income, unemployment, migration, and household expenditure on rent which is not considered in the studies in the large cities of the USA.

Matrix-based measures of health access are incomplete, and they fail to account for all aspects of the problem. Both the cumulative and gravity-based models overlook demand because they presume that services are readily available to anybody, regardless of their location or financial capacity [40]. As a result, it is difficult to compute the aggregate areal unit, which is the distance persons have to travel to reach service when examining service to population ratios [6, 41]. Access to health care is contingent not only on the availability of resources inside a community [42, 43] but also on the availability of similar resources in neighboring communities, as well as on the distance and ease of travel between them [44]. In the recent decade, the two-step floating catchment area (2SFCA) approach has become an important geographic accessibility metric, especially in the context of primary health care [45]. The inadequacies of conventional measurements of geographic accessibility inspired Luo and Wang to develop the two-step floating catchment area (2SFCA) technique. The 2SFCA technique expands upon the PPR framework by employing overlapping, mobile catchment regions to simulate and measure unrestricted healthcare access behavior [40, 46]. All services (or populations) inside the catchment are deemed accessible and equally proximate to that specific population (or service), whereas all locations outside the catchment are inaccessible [47]. The catchment size is set by a decision of maximum travel time (or distance) [6]. Despite the fact that the ability to use custom-drawn regions is the 2SFCA method’s greatest strength, this enhancement is insufficient to address the method’s other two significant flaws [40]. Before anything else, it is assumed that distance-decay within a catchment is low, which is obviously not the case in vast geographical regions with widely separated populations and, hence, relatively extensive catchments. Second, it is assumed that the size of catchments is constant across all populations and service types [48]. The 2SFCA method’s versatility makes it useful for both urban and rural communities [49].

Therefore, the two-step floating catchment area method (2SFCA) is considered in this study to analyze spatial accessibility to health facilities [6, 40, 50–57]. The service-to-population ratio for each service is computed first, and then the cumulative or gravity-based accessibility is calculated depending on the service-to-population ratio [23, 49, 58]. Earlier measures to accessibility did not take demand and supply into account. In transportation research, it is akin to the competitive access measure used to access job opportunities [59–62]. It can precisely control travel impedance, capacity constraints, and service competitiveness [63].

In transportation planning, equity is a fundamental concern, along with accessibility, as equity (justice or fairness) refers to the appropriate and fair distribution of impacts (benefits and costs) among all [64]. It has two main approaches to understanding the concept: horizontal and vertical equity [25, 65–67]. Horizontal equity is the equal distribution of benefits among all social classes. Vertical equity in public transportation requires the distribution of benefits according to the need of each social class for those services [68]. While accessibility has frequently been defined as the equity of opportunity distribution in space, the majority of health accessibility research has paid scant attention to the relationship between spatial and non-spatial variables, such as socio-economic status and access levels [69–72].

Additionally, while several academics have examined horizontal equity using the Gini coefficient and the Lorenz curve, little study on vertical equity has been undertaken. Mortazavi and Akbarzadeh (2017) considered vertical equity calculation in their research through the Spearman correlation coefficient, though it did not apply to accessibility matric [73]. For which, six cities in the state of Ohio would be studied in terms of public transportation accessibility to hospitals. In addition, the 2SFCA accessibility score is combined with household income data to provide a complete insight into the socioeconomic patterns of healthcare accessibility. Vertical equity is also assessed in this research to determine the accessibility status as transportation equity’s goal is to facilitate the services that have the need: low-income, minority, elderly, children, disabled persons, etc [24]. A comparative assessment of hospital accessibility by public transportation across multiple cities within a state enables the identification of common trends and the measurement of accessibility and equity in particular for the vulnerable population.

## Study area profile

**Fig. 1:**
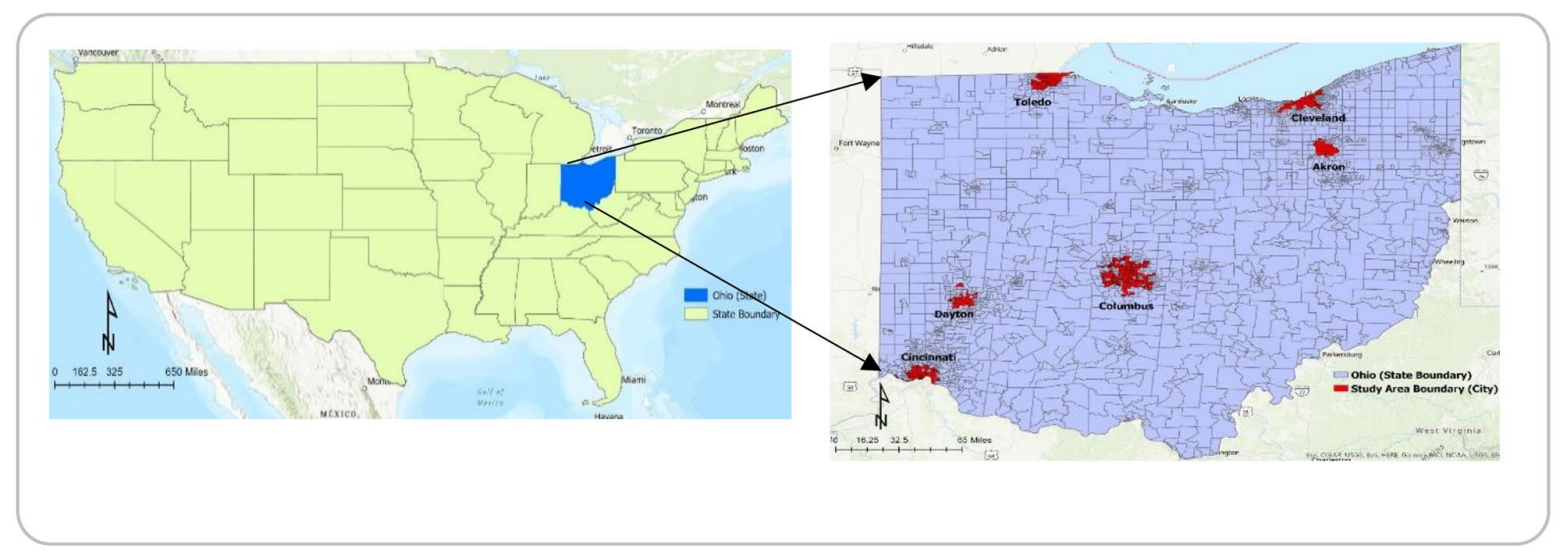
Overview of the Six Cities of Ohio

The study focuses on the six most populous cities in the state of Ohio. Columbus, Cleveland, Cincinnati, Toledo, Akron, and Dayton are these cities. These are the only cities in Ohio with a total population of more than 100,000, which is an additional notable fact about them. 20% of Ohio’s population still resides in these six cities. Figure 1 provides an overview of the six domains investigated in this study. Their primary characteristics (socio-demographic) are listed in Table 1. The transportation system is vital to provide access to everyone. If it does not work properly, it will widen socio-economic inequalities and limit people’s access to facilities like jobs and healthcare. Here, affordability contributes to measuring the accessibility to health services. Low-income people can use public buses twice than high-income people. The use of public buses is also influenced by the reliability, frequency, and convenience of bus services [74]. Ohio’s socio-economic condition can be observed by seeing the median household income. The table represents the largest cities in the state of Ohio’s demographic profile. The largest population city is below Ohio’s average median household income of $61,938 [75]. Poverty rates have also increased in Ohio that increased the demand for public transport and reduced vehicle ownership. Ohio’s population is aging, and they cannot drive and rely on public transit, especially for demand response trips. The selected six cities’ public transportation system is provided a fixed route with countywide demand response and a fixed route without countywide demand response. So, these cities have a demand for public transit to access their services (health, jobs, healthy foods, social activities, etc.). It enhances mobility for people who do not drive due to age, ability, or economic situation [21].

**Table 1:** Demographics, public transit time, and hospital bed-related information of the cities

City	Population	Pop. Density (pop/mile^2^)	Median Household Income (dollars)	Unemployment Rate (%)	Average Commute Time using Public Transport (min)	Number of Beds	Population-Bed Ratio (number of beds/1000 people)
Columbus	905,748	4132	54,902	3.8	40.5	6669	7.36
Cleveland	372,624	4796	31,838	7.4	43.7	6969	18.70
Cincinnati	309,317	3973	42,663	4.9	45.2	4457	14.41
Toledo	270,871	3365	39,155	5.4	37	1590	5.87
Akron	190,469	3075	40,281	5.5	42	2045	10.74
Dayton	137,644	2484	34,457	6	41.2	2611	18.97

The six cities vary in population, land use organization, and transportation networks. The population ranges from approximately 0.9 million in Columbus to approximately 0.1 million in Dayton, and the density ranges from 4796 to 2484 individuals per square mile [76]. Columbus has the most extensive network of high-capacity public transport systems, including bus service (run by COTA) and commuter train networks. Cleveland has BRT, Metro, Trolley, and commuter train services run by Greater Cleveland Regional Transit Authority (GRTA). Cincinnati has a metro bus and electric streetcar system for the movement of the people. Lastly, Toledo, Dayton, and Akron have only public bus services running within the city. There are two metro services run in the city of Akron.

Interestingly, average travel times on public transportation are not generally shorter in smaller urban areas. For instance, Cleveland, Cincinnati, and Akron have comparable commuting times via public transit (43.7 min, 45.2 min, and 42 min, respectively) higher than Toledo (37 min). Average public transit travel times are likely influenced by land use planning and network performance.

Consider the wide range of large cities represented in this study, as well as the fact that context-specific factors have a significant role in determining the accessibility of public transit options. For this reason, this study aims to identify the most significant hurdles to public transportation access to healthcare in Ohio’s main cities. These difficulties may be shared by multiple cities or unique to a few.

In cities of Ohio, low-income households are more likely to live in areas with higher public transportation access [77]. The outskirts of some cities, however, is nevertheless home to a significant number of low-income residents. In Cleveland, for instance, low-income individuals had more average access to employment via public transportation than their counterparts [78]. Nonetheless, a growing number of low-income households are being forced to the suburbs, partly because of rising housing costs [79]. It provides a more comprehensive view of housing affordability regions on accessibility equality, which has mostly focused on accessibility to public transportation. When discussing urban economics, some challenge the concept of trade-offs and ‘choice’ in urban transportation and housing markets by highlighting issues of supply and demand misallocation [39, 80]. Low-income people tend to travel shorter distances and rely more heavily on public transportation than those with higher incomes. The lack of options to travel long distances frequently results in shorter distances being taken. There are many examples of people who are unable to travel long distances because they lack transportation (e.g., a car or reliable public transportation) [81, 82]. However, when hospital access is taken into account, this method becomes more problematic. As a result, a large proportion of the population must travel long distances to access medical care that is more readily available elsewhere [83]. As a result, it’s critical to consider how those who rely on public transit may get to health care.

## Methodology and data sources

To calculate accessibility levels, two independent data sources were employed. American Hospital Directory (AHD) and Ohio Hospital Association (OHA) provided information on general healthcare services of Ohio (Data Link: https://www.ahd.com/). More precisely, the number of hospital beds staffed and operational in the cities of Ohio in 2019–2020 was used. AHD and OHA both list the total number of beds in each hospital. Geocoded data points were provided by the Ohio Department of Health (ODH) for each hospital in the state of Ohio. The two sets of data are then geographically combined to form a single data set. This study focuses solely on hospital services, which include access to emergency departments, major outpatient clinics, and specialist therapy [84, 85]. This type of healthcare service was chosen for two reasons: its supply is more consistent across states (no registration is required for the majority of these services, except for specialized ones), and geographic access to such services typically necessitates longer travel distances, which may require individuals to drive or take public transportation. The number of beds is utilized in this study to represent the supply at each hospital more accurately, as it represents the institution’s size and possibly the breadth of healthcare services supplied (assuming larger hospitals offer more services). Other comprehensive information, such as the number of doctors in an emergency care unit or hospital, can also be used as proxy measures; however, such data was not accessible in all cities. Table 1 summarizes the total number of available beds in all hospitals in each of the six cities, as well as the bed-population ratio (number of beds per 1,000 inhabitants). The six cities’ public transportation timetables were downloaded in the General Transit Feed Specification (GTFS) format from their respective transportation organizations. All schedules were collected for April 2022 or as close to this date as possible, depending on when the various authorities released the GTFS data. If numerous agencies serviced a single city, all agencies’ schedules with overlapping schedule dates were collected.

## Measurement of accessibility

The GTFS to Public Transit Data Model-Geoprocessing Tool of ArcGIS Pro was used for digitizing public transportation schedules. It was determined for each region how long it takes to drive from one census tract (C.T.) to another C.T. that has at least one hospital. At 10 a.m. on a typical Monday, the quickest route between C.T. centers in each city region were used to calculate trip times. Walking time from the C.T. origin (origin) to the public transportation station, waiting time, vehicle time (determined by the transit schedule), transfer time, and the distance walked from the last stop to the C.T. origin were all taken into account when determining the fastest route (destination). An off-peak level of public transit service necessitated a departure time of 10 a.m.

Spatial accessibility was then calculated using a two-step floating catchment area approach. To continue, we estimated the service-to-population ratio Vj for each hospital, taking into account the total population that can reach the service within 45 min through public transportation:

$$\begin{array}{r}{V_j} = \frac{{{S_j}}}{{\sum\limits_k {{P_k}} f({t_{kj}})}}\,\,\,and\,f({t_{kj}})\\= \left\{ {\begin{array}{*{20}{c}}{1\,if\,{t_{kj}} \le 45\,{\rm{minutes}}}\\{0\,if\,{t_{kj}} > 45\,{\rm{minutes}}}\end{array}} \right.\end{array}$$

Where Vj denotes the number of beds available per person, j signifies a healthcare service, Sj defines the service’s capacity (number of beds), Pk denotes the population in census tract k, and tkj denotes the travel time between census tract k and healthcare service j. Pk and tkj can thus be understood as the population at site k that is within 45 min of the service through transit. Second, accessibility was calculated for each census tract by adding the service-to-population ratios for the services that are within 45 min of the census tract centroid:

$$\begin{array}{r}{A_i} = \sum\limits_j {{V_j}} f({t_{ji}})\,and\,f({t_{ji}})\\= \left\{ {\begin{array}{*{20}{c}}{1\,if\,{t_{ji}} \le 45\,{\rm{minutes}}}\\{0\,if\,{t_{ji}} > 45\,{\rm{minutes}}}\end{array}} \right.\end{array}$$

Where i represents a census tract, Vj represents the service-to-population ratio for service j, and tji represents the journey time between j and I via public transportation. Thus, the accessibility metric counts the number of beds available within 45 min of each service and multiplies it by the service-to-population ratio for each service. To keep things simple, accessibility is measured in terms of beds per 1,000 persons. Census tracts are used as the study unit because it is relatively stable smallgeographic entities within counties that are delineated, and the data are easily available for the unit, which is hard If the unit goes beyond it like census block or block group, to identify and analyze. Previous studies frequently use census tracts as the basic analytical unit [84, 86–90]. This allows the findings of one accessibility study to be meaningfully compared to the findings of other accessibility studies because they all use the same spatial scale. Additionally, this makes it easier to implement longitudinal studies because the analytical unit remains stable over time. Given that the tract boundary and the city boundary are not aligned, this study solely considers the tract that falls within the city boundary. The tracts whose centroids within the city boundary are considered as census tracts of that particular city.

## Determinants of travel time threshold

This study uses a normative (i.e., prescriptive) method to figuring out what constitutes a reasonable amount of time spent traveling [85]. When it comes to “how far persons should or can reasonably travel,“ normative accessibility is typically established by a policymaker’s expectations. In this study, a decision had to be taken about what constitutes appropriate access in terms of journey time and distance. Given that the federal government requires fair access and the absence of context-specific recommendations, it is sufficient to apply a uniform criterion throughout the cities.

The criterion was established to reflect the accessibility of cities because of the prevalence of specialized healthcare in cities rather than in rural areas. Transportation planners typically use a 45-minute time limit when evaluating regional accessibility [28, 91]. These cities’ average commute times by public transportation show that the 45-minute mark is clearly visible. A healthcare accessibility criterion would’ve been wise, but the authors are unaware of any studies that have been done to advise travel time behavior and thresholds for hospitals in urban regions of the United States.

There was a sensitivity analysis done with a 60-minute threshold to find out how much the time threshold affected the results. The differences between the 45-minute and 60-minute analyses are minimal. It is essential to keep in mind that the differences in accessibility between cities are smaller when a 30-minute threshold is used, which might not fully show the differences between the areas. Given that the results were the same for both the 45-minute and 60-minute thresholds and that the 45-minute threshold used in this study is the same as the average commute time (44.8 min) for people who take public transportation in US cities [75], we are confident that the main findings discussed in this study are a good way to compare how easy it is to get to healthcare in the largest US cities.

## Measurement of equity

The socio-spatial distribution of accessibility levels was estimated using a vulnerability index derived from the demographics of the census tract’s residents. In terms of transportation, equality means giving top priority to those who are most in need [92]. Vulnerability refers to the characteristics that increase a person’s likelihood of relying on public transportation, which is a key subject of this research. According to the research, low-income individuals, recent immigrants, unemployed individuals, youth and seniors, as well as women are more likely to lack access to a private vehicle and thus rely on public transportation. The most relevant variables for the study were identified at the census tract level based on a study conducted in Canada by Foth et al. (2013) and Boisjoly et al. (2020): (i) median household income (I), (ii) unemployment rate (U), (iii) percentage of population that has migrated within the last five years (IM), and (iv) percentage of households that spend more than 30% of their total income on housing rent (R) (R). All variables were extracted from the 2016–2020 American Community Survey (ACS) 5-year data and standardized using z-scores. The final vulnerability index for a census tract is calculated as follows, where Z.X. is the z-score for variable X:

$$Vulnerability=-{Z}_{I}+{Z}_{U}+{Z}_{IM}+{Z}_{R}$$

Vertical equity was then calculated for each city to determine the distribution of health service accessibility (the number of beds in a hospital reachable within 45 min of travel time divided by the population reachable within 45 min) based on the vulnerability indicator. Using Spearman’s rank correlation index, the correlation between the vulnerability index and the accessibility levels was determined. An earlier study used Spearman’s rank correlation index to quantify the vertical equity of public transportation service distribution [73]. This method examines if census tracts with a high accessibility rank also have a high vulnerability index by assigning each one a rank for accessibility and a rank for vulnerability. Census tracts with high vulnerability (and thus a high potential for transportation and health requirements) are also likely to have the greatest accessibility, as desired from a perspective of vertical equity, as revealed by the Spearman rank correlation coefficient. The formula used to calculate the vertical equity indicator is given below.

$$Vertical Equity Indicator=\frac{{\rho }_{rAccess,rVulnerability}}{{\rho }_{max}}$$

$$=\frac{1}{{\rho }_{max}}\frac{cov\left({r}_{Access,}{r}_{Vulnerability}\right)}{{\sigma }_{{r}_{Access}}{\sigma }_{{r}_{Vulnerability}}}$$

Where ${\rho }_{rAccess,rVulnerability}$ is the Pearson correlation coefficients applied to the rank of accessibility (to low-wage jobs) and the rank of the vulnerability index, respectively, ${\rho }_{max}$ is the maximum correlation coefficient for all the United States cities, cov denotes the covariance matrix between the ranked variables, and r denotes the ranked variable’s standard deviation.

## Findings of the research

### Accessibility status across the cities with equity

**Fig. 2:**
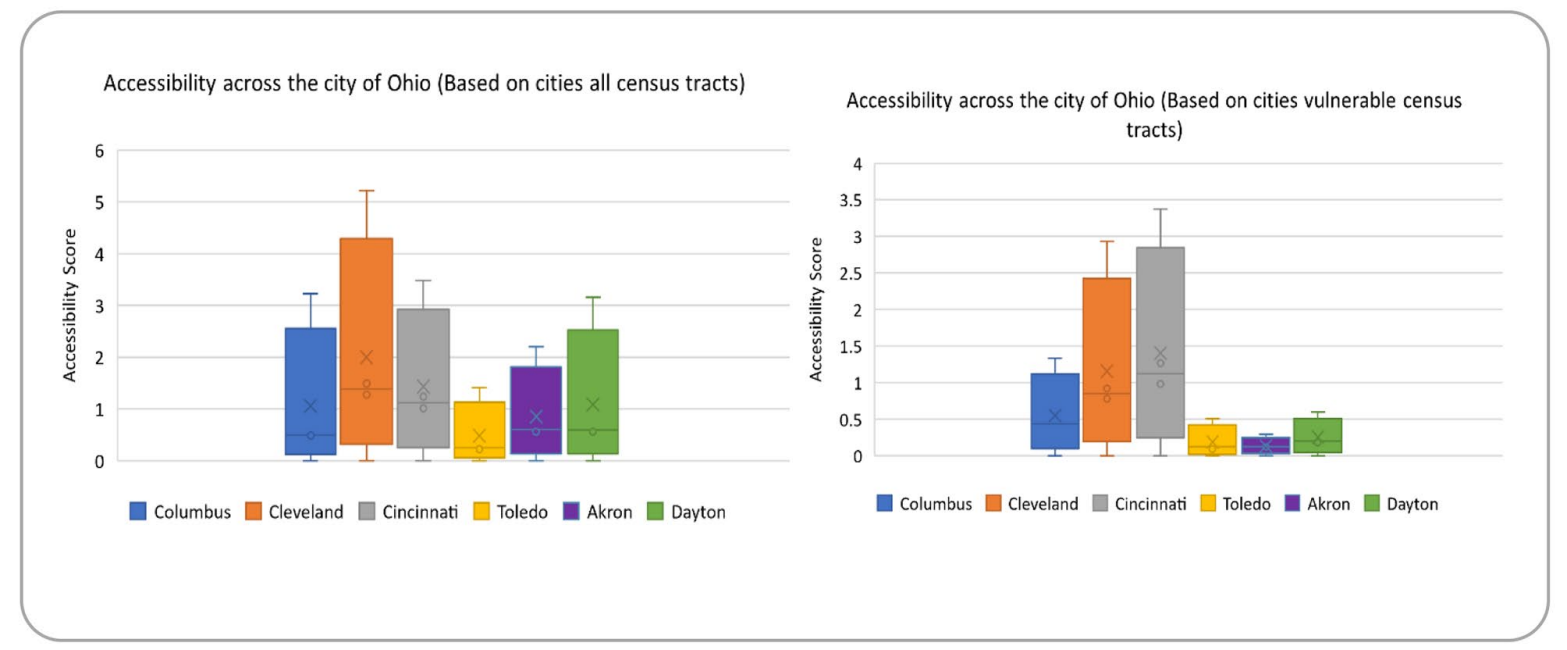
Accessibility across the cities of Ohio (number of beds/1000 people)

All census tracts and the 20% most susceptible census tracts are displayed in Fig. 2 with the mean accessibility (referred to as average accessibility) and the minimum, maximum, and standard deviation. In order to proceed, it is important to understand that the minimum accessibility requirement for all metropolitan statistical areas (MSAs) is null, which means that at least one census tract is inaccessible. As a result, this study focuses on the overall accessibility of hospitals in each city area, but future studies could look at regional differences in this regard. While the second and third largest cities in terms of population, Cleveland, and Cincinnati respectively, have high standard variation and are among the 20% most vulnerable census tracts. The high maximum accessibility scores they have also suggest large variations between census tracts.

**Fig. 3:**
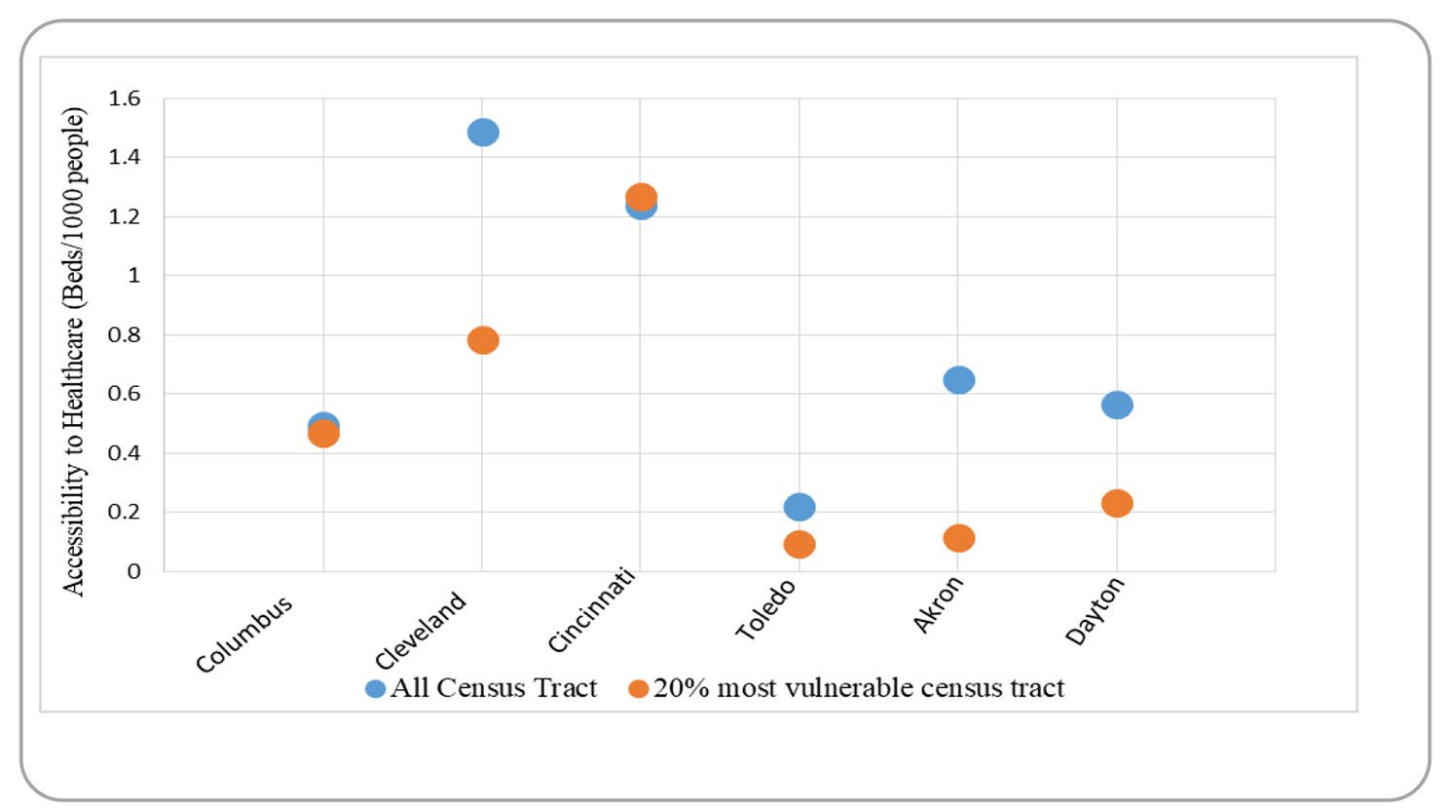
A comparison of accessibility standards in Ohio’s six cities

Figure 3 compares the average accessibility to healthcare between the city people and 20% most vulnerable people in census tracts. Columbus, the largest city in the state of Ohio in terms of population, has the second-lowest average level of healthcare accessibility, while Toledo has the lowest level of healthcare accessibility. On the other side, Cleveland and Cincinnati have the highest levels of accessibility, with Dayton and Akron coming in close after.

It’s worth noting that Cleveland, and Cincinnati, which have the highest average accessibility to healthcare for all citizens, also have the most beds per capita (17.51, and 14.22, respectively – see Table 1). In comparison, Columbus and Toledo have the lowest bed-to-population ratios (8.25 and 5.87, respectively), resulting in low accessibility levels. Interestingly, Dayton has higher beds per capita ratio (18.58) than Akron (10.74) but has a lower accessibility rate than Akron. It indicates that, while the quantity of supply is a significant determinant of accessibility, other factors such as the spatial distribution of hospitals and the performance of the public transportation system also come into play when examining accessibility to healthcare services, two of which are discussed in the following section.

For fairness, vulnerable census tracts need to have a higher rate of access to healthcare than the area as a whole. But it is not one of these Ohio cities. The vulnerable tracts have mostly seen lower accessibility compared to all the tracts. This shows an inequitable distribution of accessibility in terms of vertical equity since underprivileged communities are more likely to rely on public transportation to obtain healthcare services. This situation is most pronounced in Cleveland, where inhabitants of vulnerable census tracts had access to 91% fewer services than the average (accessibility values of 1.49 and 0.78, respectively). Additionally, these people have the lowest accessibility value across all areas except Cincinnati. Cleveland, Akron, and Dayton have shown a higher difference in access between the vulnerable and other groups of census tracts. It is clearly identified from Fig. 2 that the discrepancy between the access of vulnerable tracts in comparison to others is quite high among these three cities (Cleveland, Akron, and Dayton show 91%, 81.25%, and 59% less access to the health care services to the 20% vulnerable tracts). In comparison, Only Cincinnati has slightly higher accessibility levels to the 20% most vulnerable census tracts compared to all census tracts (accessibility values of 1.27 and 1.24, respectively).

**Fig. 4:**
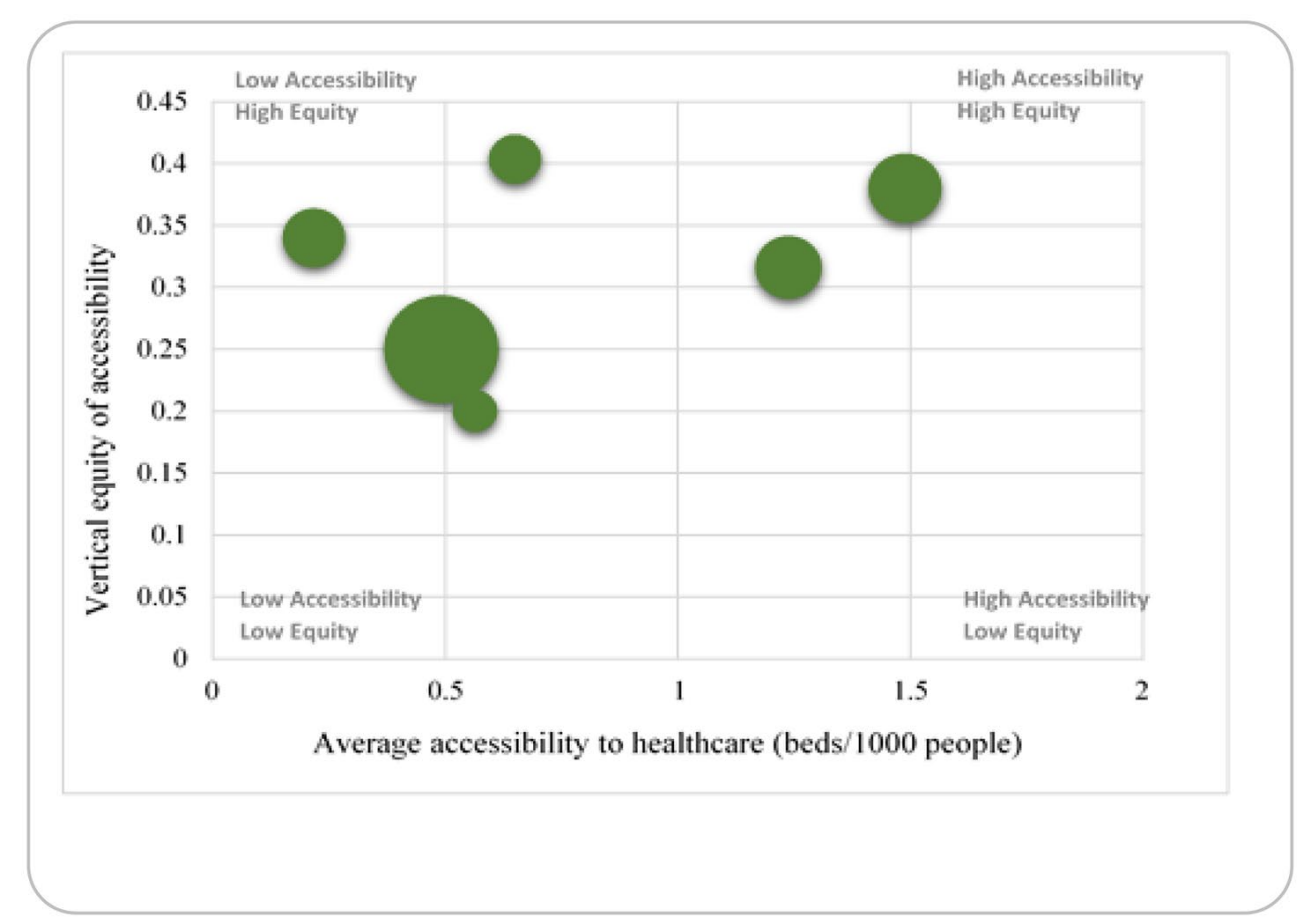
Accessibility and vertical equity in the six cities of Ohio

A vertical equity indicator was created for each city in Ohio using Spearman’s rank correlation index to further study the socio-spatial distribution of accessibility across the cities. The six major city areas are compared in terms of average healthcare accessibility (x-axis) and vertical equity of healthcare accessibility (y-axis) in Fig. 4. (y-axis). The size of the circles is related to the population of the city. A city in the upper right corner illustrates the ideal situation: an area with high overall levels of access to hospital beds and where this access is evenly distributed across various socioeconomic categories, i.e., residents in vulnerable census tracts often have more accessibility. Surprisingly, Columbus is among the cities with the lowest vertical equity index as well as lowest accessibility index to healthcare, given that vulnerable census tracts in the city are quite similar access compared to the average access value of the region. In fact, Columbus is the largest among all the cities of Ohio, which also provides proof of having not good access and equity rate for the public transport user. It demonstrates the difficulties of serving vulnerable census tracts when the average level of healthcare accessibility is already low. On the other hand, Cleveland is the only city in the state of Ohio with a moderate access and vulnerability index rate since vulnerable census tracts have slightly higher accessibility than the region’s average. All the other cities except Columbus, Dayton, and Toledo have either better equity than accessibility or the reverse situation identified in Fig. 4. Columbus, Cleveland, Cincinnati, Toledo, Akron, and Dayton are the six cities in the state of Ohio with a population of over 100,000, and nearly five of them have indications of poorer accessibility or equity to the vulnerable census tracts where the majority of public transport users reside.

Additionally, it appears that the bigger cities of Ohio (Columbus) perform poorly on a vertical equity basis. The smaller cities (Toledo, and Dayton) have the lowest vertical equity indices. The suburbanization of poverty and the concentration of healthcare services are two possible explanations for this trend. Compared to larger cities, such as Columbus, the vertical equity in such small cities is high. As a result, the bulk of these cities’ vulnerable census tracts are located in or near the downtown area, which is home to numerous hospitals. There is a high vertical equity index for disadvantaged census tracts in these cities because they have more healthcare access than the rest of the area does.

In general, the data show that larger cities (excluding Columbus) do better in terms of vertical equity and average accessibility to healthcare services. When it comes to accessibility, census tracts in smaller cities suffer from poor vertical equity and lower average access. Low bed-to-population ratios (in Columbus and Toledo) could be to blame, or the difficulty of serving a widely dispersed populace could be to blame. As a result, the study’s findings imply that greater efforts are needed in large urban regions like Columbus to increase healthcare accessibility particularly for vulnerable census tracts.

### Socio-spatial pattern of accessibility

**Fig. 5:**
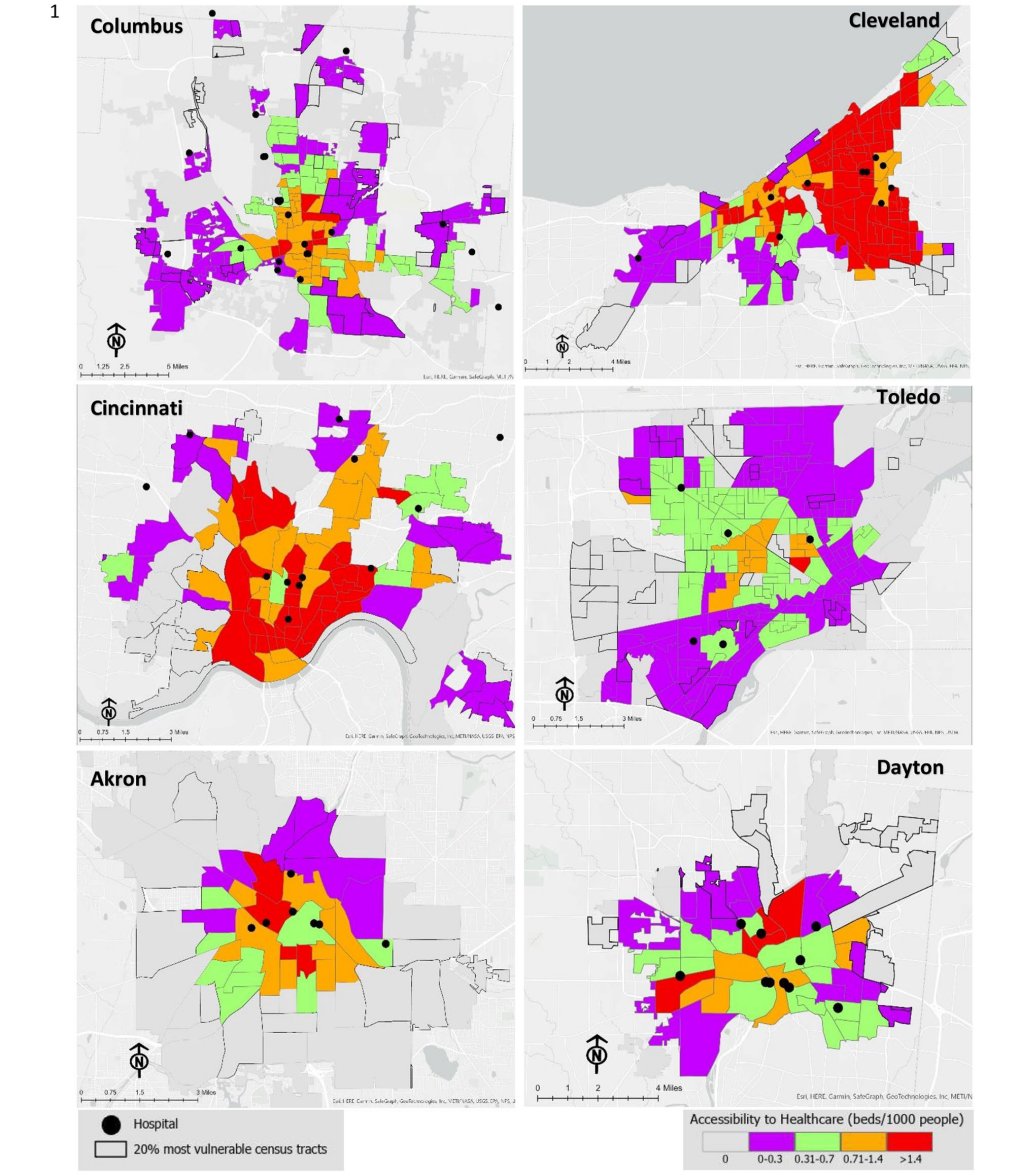
Accessibility to healthcare in the six cities of the state of Ohio

Specifically, this section examines the important land use and transportation issues that have a negative impact on access to healthcare in certain locations, particularly for vulnerable groups. When looking at the maps in Fig. 5, most of the healthcare facilities are creating a cluster in the center of the cities. On the contrary, the vulnerable tracts of the cities are mostly seen on the edge of the city boundary, which is also shown on the map by the black outline. The peripheries of the cities experience low-level accessibility due to a lack of public transport services and hospitals. All the cities have some tracts that have no access to the hospital and in most cases, the vulnerable groups live there. These six cities of Ohio highlight several issues regarding access to healthcare.

Among the three largest cities in Ohio, Columbus has shown less accessibility to healthcare by many tracts compared to the other two (Cleveland and Cincinnati) and has less comprehensive public transport service, especially for the vulnerable group. Columbus’s fringe area has shown a low level of accessibility by public transit service. Although Cleveland has better access to healthcare services but gives an uneven illustration of healthcare services as most of the hospitals are on the east side compared to the west (only 2 hospitals). In Cincinnati, the southern part has no healthcare facility, and even the public transit network does not fulfill the demand for those tracts to access the services. It shows inequality in the distribution of healthcare services to different tracts. Even though the region’s accessibility is overall rather good, the in-depth analysis of accessibility indicates that it is unevenly distributed.

On the other hand, Toledo, Dayton, and Akron fully show the centralized healthcare service, whereas Toledo has fewer hospitals than other cities. Even most of the tracts of Toledo showed the lowest level of accessibility due to the poor service and connection system of the public transit. It clearly shows the public transport system of Toledo is not developed in a proper way that can serve their people. In Akron and Dayton, most of the vulnerable tracts remain on the fringe of the city boundary area, and due to the shortage of better public transit service, they are fully deprived of the healthcare service in the center of the city.

A common notion of accessibility is seen among these cities, which reveals that all the cities have provided evidence of uneven distribution. Even in the smaller cities (Akron, Dayton), the situation is not different. As the most disadvantaged census tracts in these locations are concentrated in the outskirt, resulting in a low vertical equity index, it is important to look into how public transportation may be made easier for the vulnerable group to access healthcare facilities. The majority of vulnerable census tracts are located outside of the city core, which is home to a few hospitals, according to vertical equity analysis. Census tracts with high levels of vulnerability are less accessible. Consider vulnerable census tracts’ locations while decentralizing healthcare services and increasing public transportation to vulnerable census tracts in order to ensure accessibility.

In the accessibility analysis map, it is seen that even though some tracts have several numbers of hospitals, the accessibility is not very high in those tracts. The reason behind the low accessibility is either less services provided by hospitals (the number of beds is not high) or the public transport facility is not developed in a better way. For example, Toledo has several tracts and even vulnerable tracts in the center, even though hospitals are not far from that tract. The reason behind low accessibility is the poor connectivity of public transit services. Expanding services in the region (by building new hospitals or adding beds to existing ones) or improving public transportation access to neighboring hospitals, particularly those that now face minimal competition, could both help improve access from these census tracts of the city. Increasing the accessibility of healthcare services via public transportation would also be a benefit to the region.

## Discussion and conclusion

US metropolitan area indicates that effective public transportation is absent in suburban area due to low-income households and this substantial poverty also become a barrier to providing appropriate access to healthcare services in the US [93, 94]. This type of poverty problem is not limited to here but also noticed in middle- and low-income nations worldwide notably Latin America [95–99]. Hence, this study assessed the spatial accessibility of general medical and surgical hospitals throughout six cities in the state of Ohio, considering both the trip times by public transport and the service-to-population ratio, with the number of beds serving as a proxy for the level of service to observe this situation. A benchmarking methodology is used to examine the accessibility of healthcare providers within a 45-minute drive of the research location. Interestingly, the findings show that, other than Cincinnati, the socio-spatial distribution of access to healthcare is not vertically egalitarian in any other cities. Cities with inadequate accessibility are home to 20% of the most vulnerable residents. As a result, those with the greatest requirements also have the most difficulty accessing services. Almost all of the cities’ urban regions have been affected by the current state of affairs.

Consequently, vulnerable census tracts in these cities have the lowest levels of public transport accessibility to healthcare services in Ohio. This is largely attributable to the inaccessibility of hospitals located in the periphery by public transportation and the high proportion of vulnerable households in the inner suburbs of the regions because of the suburbanization of poverty that many of the world’s largest cities have been experiencing.

Increasing public transportation-based access to healthcare services in the suburbs could benefit everyone, but it would be especially beneficial to the most vulnerable members of society. Better public transportation access to healthcare services in the United States and Canada has been associated with increased usage of healthcare services, according to research conducted in these countries [100, 101]. Despite this, little is known about the effects of public transportation on the way people use healthcare. When it comes to overcoming obstacles to healthcare access, Syed et al. (2013) conducted an in-depth evaluation of the literature [101]. They found that this is an important area of research that needs to be done. Insufficient accessibility and accessibility gaps can exacerbate socioeconomic and health disparities [31]. For instance, Linden, a neighborhood in Columbus, Ohio, confronts numerous obstacles due to its limited access to vital resources such as employment and healthcare facilities. Linden’s median household income is less than half that of the city, and unemployment rates in some portions of the neighborhood exceed 15% [102, 103]. In addition, the infant mortality rate in South Linden is close to 26, which is around four times the national average [102, 104]. The city of Columbus is working with the city’s primary public transportation agency, the Central Ohio Transit Authority (COTA), to introduce new public transportation options to help more people take advantage of the city’s many opportunities.

Various indices can be used to measure healthcare availability in different locations, as demonstrated by the study. Vulnerable census tracts tend to be located in less accessible cities with lower levels of accessibility and vertical equity. In order to provide context-specific advice and better understand the socio-spatial distribution of accessibility to healthcare services, it is required to move beyond these indicators, as described in the preceding section. Many hospitals in Cincinnati’s perimeter have little competition, and improving public transportation to these facilities will have a substantial impact on strengthening vertical equity and average accessibility. According to in-depth research of Akron’s city, there are notable differences between the city’s core (which is easily accessible) and its perimeter (which is not). Increasing public transit access to hospitals in the periphery could help to ensure a more even distribution of patients.

Despite the fact that identical patterns can be observed in other urban areas, context-specific interventions are required to expand healthcare access. Therefore, greater efforts are needed to analyze how healthcare access is considered in the establishment of public transportation and health policy in diverse regions. Although health and transportation are increasingly intertwined [105], policymakers and planners are still primarily focused on the benefits of active transportation, physical activity, and noise and pollution exposure [106, 107]. Access to healthcare services via public transportation has been studied extensively since schedule data was made available to everyone in the early 21st century [23, 63, 84, 91, 108, 109]. However, little is known about how these criteria are included in planning methods, and more research is needed to determine the challenges and potential for collaboratively addressing public transportation and healthcare access planning. Also, future studies should employ regression modeling to figure out the factors affecting accessibility.

The research has some limitations. First, hospital accessibility was established at the census tract level by calculating travel times using the centroid. Therefore, travel times to hospitals may be underestimated or overestimated, particularly for large census tracts (mainly on the region’s periphery). In most cases, travel time has little effect on accessibility calculations. In a few instances where the hospital lies at the edge of a large census tract, the travel time estimated using the census tract’s centroids and the hospital’s actual location diverge significantly. As large census tracts are typically located in the periphery, where public transport is limited, the impacts (overestimation or underestimation) on accessibility are limited to the few census tracts surrounding the hospital, the others being more than 45 min away regardless of the method used to calculate travel time. The findings of this study reflect cities access patterns. To establish peripheral accessibility, more studies with a higher spatial resolution could be conducted. Our research does not incorporate travel time dependability. The GTFS data offers transit schedules that account for traffic congestion, however daily traffic circumstances vary. Future measures should take these distinctions into account. This study focuses on access to general medical and surgical facilities of the United States, but future research might concentrate on primary care. Accessibility to healthcare during emergencies was not addressed in this study because it required more exact data on mode availability and shorter journey time thresholds, which were not the focus of the investigation. In our study, we used a 45-minute threshold to derive accessibility measures. Future studies may use different thresholds or apply other accessibility measures, such as gravity-based measures, if the data required to generate such measures are available for all studied regions from an origin-destination survey that includes healthcare services. The disparities in health outcomes can be attributed not only to limited spatial accessibility to healthcare providers, but also to factors such as unaffordability (e.g. lack of medical insurance, low income), and lack of trust in the healthcare system, particularly among African-Americans. This distrust can be traced back to past incidents such as the Tuskegee Syphilis Study [110].

The method used in this study could be used as a basis for future research that looks at how different thresholds affect real access to healthcare. In other words, at what point does the time it takes to get somewhere by public transportation make it hard to get healthcare? It would also be useful to do these kinds of studies in different cities to find out if different factors in each setting lead to different results.

## Data Availability

All the secondary data are available on the website (census bureau). Link: https://data.census.gov/all?q=employment. https://www.ahadata.com/topics/hospital-data.
